# The evaluation of serum lipid profile and apolipoprotein C-1 in the Iranian patients of Oral Squamous Cell Carcinoma

**DOI:** 10.37796/2211-8039.1349

**Published:** 2022-09-01

**Authors:** Walaa Alazzawi, Zahra Shahsavari, Hanieh Babaei, Hadis Firouzpour, Abbas Karimi, Afsaneh Goudarzi

**Affiliations:** a Department of Clinical Biochemistry, School of Medicine, Shahid Beheshti University of Medical Sciences, Tehran, Iran; b Student Research Committee, School of Medicine, Shahroud University of Medical Sciences, Shahroud, Iran; c Oral and Maxillofacial Surgery Department, School of Dentistry, Tehran University of Medical Sciences, Tehran, Iran

**Keywords:** Oral squamous cell carcinoma, Serum lipid profile, Serum ApoC1

## Abstract

**Background:**

The key role of apolipoprotein C-1 (ApoC-1) is reported in breast, pancreas and lung cancer. However, no information is available on potential difference of ApoC-1 between OSCC patients and healthy individuals. This work aimed to examine the serum ApoC-1 level as well as lipid profile values between OSCC patients and healthy control groups.

**Material and methods:**

In this study, 44 blood samples from 22 OSCC patients and 22 healthy individuals were collected to determine the values of lipid profile and ApoC-1 concentration using colorimetric method and Enzyme-Linked Immunosorbent Assay (ELISA), respectively.

**Results:**

A significant decrease in serum lipid profile and ApoC-1 concentration was observed between OSCC and healthy control groups (P = 0.001).

**Conclusion:**

Our results confirm the previous findings on the significant differences of lipid profile between OSCC and controls, also show the lower serum level of ApoC-1 in OSCC as compared to the controls. Future studies would further elaborate the association of ApoC-1 with OSCC.

## 1. Introduction

Despite recent progress in therapeutic strategies, the outcome of Oral Squamous Cell Carcinoma (OSCC) patients has not improved yet [1]. OSCC accounts for more than 90% of all neoplasms in the oral cavity with a 5-year survival rate less than 50% [2]. The diagnosis of OSCC occurs at late stage and is more common in developing than developed countries [3]. The major cell components are lipids which have indispensable roles in growing and dividing of normal and malignant tissues [4]. High rate of lipid peroxidation releases peroxide radicals which attack essential components of the cell membrane thus increase the demand on lipids including total cholesterol, lipoproteins and triglycerides for synthesis of new membrane [5]. Recent studies have shown the association of altered lipid profile with head and neck squamous cell carcinoma, breast cancer, ovarian tumours and colorectal cancer [6–9]. Apolipoproteins are key proteins in regulation of lipoprotein metabolism, their basic functions are lipid delivery, the activation or inhibition of enzymes involved in metabolism of lipoproteins and as ligands to recognize the lipoproteins’ receptors [10]. Recently, published results support abnormal expression of apolipoproteins in tumours which is related to the prognosis of patients [11]. Hence, the attention has focused on the association of apolipoprotein family in tumours’ incidence and progression. ApoC-1 which belongs to apolipoprotein family contributes to the Chylomicron, VLDL and HDL-C metabolism [12] and its involvement in progression of several diseases including diabetes, polycystic ovary syndrome, Alzheimer’s disease and glomerulosclerosis has been shown [13–15]. Although numerous studies revealed that ApoC-1 is associated with the tumour progression but the exact underlying mechanism and signaling pathway of ApoC-1 in tumours is still unclear [16–18]. The correlation of high tissue expression and high preoperative serum concentration of ApoC-1 with poor prognosis of pancreatic cancer patients has been reported by Li and colleagues [18]. Also, the diagnostic ability of overexpressed ApoC-1 has been reported to distinguish triple negative breast cancer (TNBC) from non-TNBC (NTNBC) and is considered as a potential prognostic biomarker for TNBC [19]. The upregulated mRNA and protein expression levels of ApoC-1 in prostate cancer tissue is correlated with survival of prostate cancer cells by inhibiting apoptosis, also the increased serum level of ApoC-1 is associated with the prognosis of prostate cancer patients [17]. The significant decrease in serum levels of ApoC-1 has been reported in colorectal cancer, NSCLC [11,20], papillary thyroid carcinoma [21] and child nephroblastoma [22]. However, the serum level and role of ApoC-1 in OSCC remain elusive. In this experimental study, we sought to investigate and compare the serum lipid profile and ApoC-1 levels of OSCC patients with healthy individuals.

## 2. Materials and methods

### 2.1. Subjects

The serum samples of 22 patients diagnosed with OSCC who had surgery from January 2019 to April 2021 were provided from a Pathology laboratory (School of Dentistry, Cancer Institute Hospital) and two hospitals (Bahman and Shariati hospitals) of Tehran, Iran. The clinicopathological description of study groups are shown in Table 1. All patients had a histologically biopsy proven diagnosis for OSCC, belonged to an age group of 38–84 years. Patients who had other diseases or were under treatment with medications that could affect their lipid profile (diabetes, liver failure or thyroid disorders) were excluded. 22 healthy subjects of control group were age- and gender-matched without any history of major illness in the past. The tumours were graded histologically to grade I (well-differentiated), grade II (moderately differentiated) and grade III (poorly differentiated) based on WHO criteria. Tumour staging has been performed according to the American Joint Committee on Cancer (AJCC). Clinical information including age, gender, tumour location were obtained from the information file of patients. This study was performed at Clinical Biochemistry department of Shahid Beheshti University of Medical Sciences (SBMU) under ethical approval of SBMU: IR.SBMU.MSP.REC.1400.168.

### 2.2. Serum lipid profile assessment

The lipid profile tests measure serum total cholesterol (TC), triglyceride (TG), HDL-C, LDL-C, and VLDL. After a 12–14 h overnight fasting, 5 ml of blood sample was collected under aseptic precautions in Serum Separator Tubes. To prepare the serum, the collected blood samples were clotted for 1 h and then centrifuged for 10 min, 3000 rpm at room temperature. The serum samples were stored in the freezer −80 °C until measurements. The serums were then analyzed for lipid profile tests by the commercially available kits, Cholesterol (Pars Azmoon, Iran), Triglyceride (biorexfars, Iran) and HDL-C (PishtazTeb, Iran) using a biochemistry autoanalyzer (BS-200 E, mindray, USA). The levels of LDL-C and VLDL were calculated by the following formula:


LDL-C=(0.94×Total Cholesterol)-(0.94×HDL-C)-(0.19×Triglyceride)[
23].

VLDL=TG/5[
24].

### 2.3. Serum ApoC-1 measurement

Concentrations of ApoC-1 in serum samples of OSCC patients and healthy individuals were determined by commercially ELISA kit (Invitrogen, # EHAPOC1) following the manufacturer’s protocol. In brief, the wells in ELISA plate were coated with 100 μl of standards (concentrations from 3.2 to 50,000 pg/ml of ApoC-1) and samples (diluted with sample diluents buffer in a proportion of 1/10,000) and incubated 2.5 h at room temperature. Next, plate contents were discarded and 300 μl of 1X wash buffer was used to wash four times each well. Then, 100 μl of the biotinylated human anti-ApoC-1 conjugate was added to each well. The plate was incubated 1 h at room temperature with gentle shaking and then the contents were removed and washed four times with 1X wash buffer. After washing, 100 μl of the Streptavidin–HRP solution was added to each well and incubated 45 min at room temperature. Next, the solution was removed and each well was washed four times with 1X wash buffer. After washing, 100 μl of TMB solution was added to each well and then incubated 30 min at room temperature in the dark with gentle shaking (The substrate began to turn blue). Next, the reaction was stopped by adding 50 μl of the stop solution to each well. The plate was immediately read at 450 nm. The standard curve was generated using curve-fitting software and Apo-C1 concentrations of unknown samples were determined from this standard curve.

### 2.4. Statistical analysis

Data analysis was performed using GraphPad Prism version 6 and data are expressed as mean ± standard deviation (SD). Statistical analysis was done using the ANOVA test and unpaired t-test. Correlation coefficients were calculated using the two-tailed Pearson’s correlation analysis. In all the above tests, P-value < 0.05 was taken to be statistically significant.

## 3. Results

### 3.1. Decreased serum levels of lipid profile in OSCC patients compared to healthy individuals

**Fig. 1:**
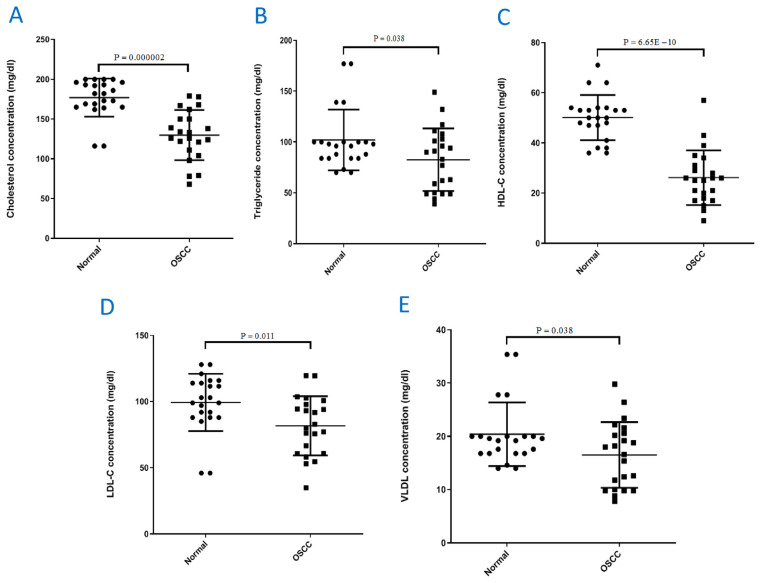
Comparison of lipid profile levels between OSCC patients and healthy individuals. (A) Cholesterol, (B) Triglyceride, (C) HDL-C, (D) LDL-C, (E) VLDL. P < 0.05 is significant.

In this study, a total of 11 male and 11 female patients with median age of 63 years were included, of them 4.54% patients had lesion on oral cavity, 9.09% on maxilla, 22.72% on tongue, 4.54% on mouth floor, 40.9% on lip/buccal mucosa, 9.09% on palate and 9.09% on mandibule. 59.09% patients were graded as grade I, 31.81% grade II and 9.09% grade III. To compare the serum lipid profile between the OSCC patients and controls, the mean and P-values of all the serum lipid profile values were calculated and compared. A Significant decrease in serum cholesterol (P = 0.000002), triglyceride (P = 0.038), HDL-C (P = 6.65E-10), LDL-C (P = 0.011) and VLDL (P = 0.038) concentrations was found in OSCC patients compared to the healthy individuals (Fig. 1) which confirmed previous reports by [4,25]. Next, we compared the values of all the lipid profile with the clinical and pathological features of patients including tumour subsite, tumour grade and stage (Tables 2–4). To this end, OSCC patients were divided in three groups of well differentiated (grade I), moderately differentiated (grade II) and poorly differentiated (grade III). A significant cholesterol reduction was observed between grade I group and healthy individuals (P = 0.0001), also a significant lower value of HDL-C was observed between healthy individuals, grade I (P = 4.9E-8), grade II (P = 0.00005) and grade III (P = 0.039) groups (Table 2). To understand whether the values of lipid profile differ with healthy individuals depending on the site of tumour, we divided patients into different groups based on the tumour location and compared the serum lipid profile of these groups with controls, the obtained results are shown in Table 3, for instance lower triglyceride levels in OSCC patients compared to control group was significant only in patients with Maxilla (P = 0.022) and oral cavity mucosa tumours (P = 0.019) (See Table 3). Subjects with stage II (P = 0.019), III (P = 0.007) and IV (P = 0.0002) had lower cholesterol level compared to controls; the HDL-C was lower in all the stages I (P = 0.008), II (P = 0.000003), III (P = 0.0001) and IV (P = 0.00001) compared to the healthy individuals; also LDL-C was lower only in stage IV compared to controls (P = 0.043) (See Table 4).

### 3.2. Decreased ApoC1 serum concentration in patients with OSCC compared to healthy individuals

**Fig. 2:**
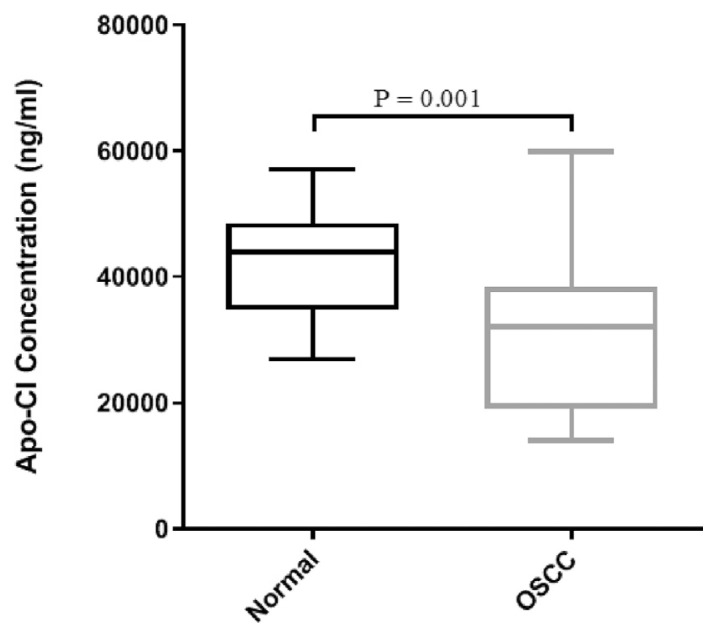
Comparison of ApoC-1 levels between OSCC patients and healthy individuals. Distributions of ApoC-1 concentrations in OSCC patients and controls using box-plots. P < 0.05 is significant.

In order to investigate the clinical value of ApoC-1 serum level, first we compared the serum level of ApoC-1 between OSCC patients and controls to see whether it is altered in OSCC or not. As indicated in Fig. 2, the results revealed the significant decrease in serum ApoC-1 of OSCC patients as compared to healthy individuals (P = 0.001).

### 3.3. The serum ApoC-1 is not predictive of OSCC staging, grade and tumour size

**Fig. 3:**
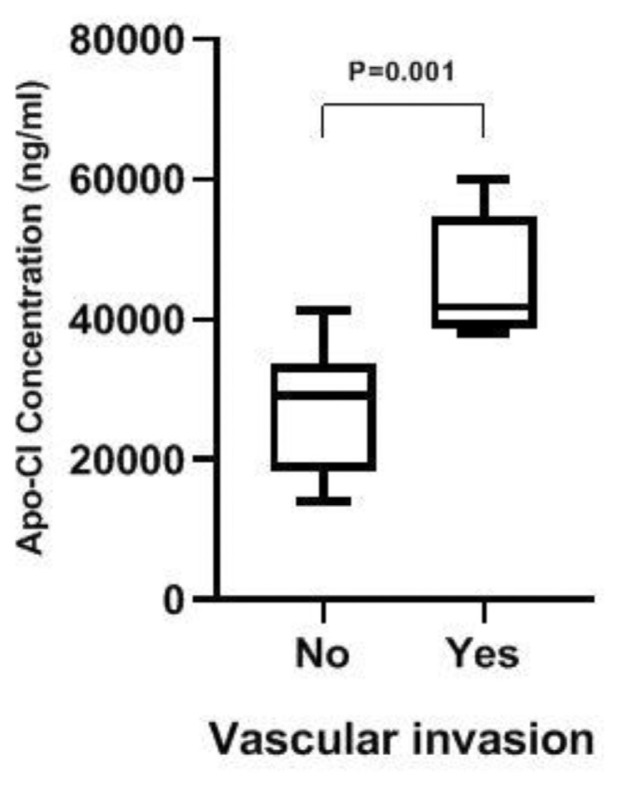
Comparison of ApoC-1 levels between OSCC patients with and without vascular invasion. Box plot showing the distribution of ApoC-1 concentrations in two groups of OSCC patients defined as Yes = with vascular invasion, No = without vascular invasion. . P < 0.05 is significant.

The observed alteration in ApoC-1 serum level of OSCC patients motivated us to examine its correlation with clinical and pathological features of patients. Neither tumour size (P = 0.67) nor grade (P = 0.7) were significantly correlated with serum ApoC-1 concentration (Table 5). Interestingly, we found that for patients with vascular invasion, the ApoC-1 level is statistically higher than for those without vascular invasion (Fig. 3 and Table 5).

## 4. Discussion

Lipids play important roles as energy storage, structural component of cell membranes and signaling processes [25,26]. Tumour cells need high level of lipids to fulfill the requirements of cell rapid proliferation and growth, thus exhibit changes in lipid metabolism pathways. To this end, tumour cells show increased lipid uptake, endogenous de novo lipogenesis, oxidation of fatty acids and cholesterol accumulation [27]. Lipid metabolism deregulation is one of the cancer hallmarks and has been considered as promising therapeutic targets [27]. For instance the over-expression of Fatty Acid Synthase (FASN) which is a key lipogenic enzyme has been reported to be associated with proliferation, chemoresistance and poor prognosis of ovarian cancer and FASN inhibitors have been shown to promote cancer cell apoptosis [28]. Therefore, the decrease in blood lipids in some cancers can be due to the effects of cancer cells’ metabolism, disruption of the metabolic pathways of lipids, increased lipid peroxidation and antioxidant vitamins. To compensate for their lipid deficiency, cancer cells increase the lipogenesis or meet the need for the breakdown of blood lipoproteins such as HDL-C, LDL-C and VLDL [7]. Lipoproteins are in charge of delivering endogenous and exogenous lipids to the tissues [29]. ApoC-1 as a surface component of lipoproteins including chylomicron, HDL and LDL is mainly expressed in liver and its oncogenic role has been reported in pancreatic, breast and lung cancer [18,30,31]. Several studies declared that ApoC-1 mediates cell survival and the knockdown of ApoC-1 significantly has decreased proliferation of pancreatic cancer cells and induced apoptosis [18]. This study aimed at investigating both the serum lipid profile and ApoC-1 levels in OSCC patients and healthy individuals. We confirm the findings of previous studies on lower serum lipid profile values including TG, cholesterol, HDL-C, LDL-C and VLDL of OSCC patients compared to the controls [4,25]. Except to HDL-C, we found a progressive decline in all lipid profile values as stage increases. Additionally, the serum mean value of cholesterol compared to healthy individuals is significant only for stage III and stage IV. Regarding the histopathological grades, among all measured lipids only cholesterol decreases as grade increases. This is the first report that provide evidence on the significant differences of serum ApoC-1 between OSCC and healthy individuals. We found the lower serum ApoC-1 level in OSCC compared to the healthy individuals whereas Song et al. recently found that the serum level of ApoC-1 was increased in patients with TNBC compared to non-TNBC [30]. On the other hand, Li and colleagues reported reduction of serum ApoC-1 level of pancreatic cancer patients after surgery compared to the preoperative serum confirming that pancreatic cancer cells secrete ApoC-1 [18]. To unravel the reason behind lower serum level of ApoC-1 in OSCC patients compared to the healthy individuals, one need to investigate and compare the ApoC-1 tumour expression with normal oral mucosa tissue. The OSCC patients with vascular invasion showed significantly higher ApoC-1 level as compared to those without invasion. Neither grade nor stage was associated with significant decrease of serum ApoC-1 level. Taking into account the location of studied malignancy, saliva ApoC-1 is more relevant than serum and the recent work by Hirtz et al. on saliva revealed that ApoC-1 up-represented in OSCC without lymph node metastasis vs. OSCC_FREE and OSCC with lymph node metastasis [32]. A future study to measure ApoC-1 on saliva, serum and tissue of the same OSCC patients and comparing it with healthy individuals is needed.

## 5. Conclusion

Decreased lipid profile and ApoC-1 levels in our study may indicate a correlation between these factors and the progression of OSCC. The main limitation of this study was the evaluation of ApoC-1 in the serum and tumour tissue of the same patients which could potentially enable us to hypothesize whether OSCC tissue expresses and secretes ApoC-1 in the serum or not. Additionally, since the apolipoprotein E (ApoE) and ApoC-1 genes belong to the same gene cluster, it would be very informative to correlate the decrease in plasma cholesterol with apoE in future research. Further investigation to unravel its prognostic importance is needed and would potentially lead to a new therapeutic target for OSCC patients.

## Figures and Tables

**Table 1:** Clinicopathological characteristics of OSCC patients and healthy controls.

Characteristics	OSCC patients (n = 22)	Healthy controls (n = 22)
**Age Mean (y)**	63	62.4
**Age (y)**		
≤60	7	10
>60	15	12
**Sex**		
Female	11	8
Male	11	14
**Grade**		
Grade I	13	–
Grade II	7	–
Grade III	2	–
**Stage**		
I	3	–
II	5	–
III	6	–
IV	6	
**Tumour size**		
≤3.5 cm	10	
>3.5 cm	10	
**Death**		
Yes	1	0
No	21	22
**Vascular invasion**		
Yes	6	–
No	8	–
**Perineural invasion**		
Yes	7	–
No	7	–
**Tumour subsites**		
Oral cavity	1	–
Tongue	5	–
Mouth floor	1	–
Lip/Buccal mucosa	9	–
Maxilla	2	–
Mandible	2	–
Palate	2	–

**Table 2:** Comparison of lipid profile and tumour grade between OSCC patients and healthy controls.

	Cholesterol	Triglyceride	HDL-C	LDL-C	VLDL
**Healthy Controls**	164.61 ± 29.11	104.08 ± 31.81	47.00 ± 9.50	90.58 ± 24.00	20.81 ± 6.36
**Tumour histopathology**					
Grade I	122.54 ± 34.05	79.92 ± 35.05	23.61 ± 11.70	77.80 ± 23.67	15.98 ± 7.01
Grade II	141.28 ± 25.89	87.57 ± 26.41	29.00 ± 9.50	88.91 ± 19.45	17.51 ± 5.28
Grade III	136.5 ± 36.06	82.00 ± 26.87	32.50 ± 9.20	82.18 ± 30.36	16.40 ± 5.37
*p* value* comparison of lipid profile of OSCC and healthy controls					
Grade I* Healthy controls	0.0001	0.351	4.9E-8	0.057	0.351
Grade II* Healthy controls	0.008	0.152	0.00005	0.388	0.152
Grade III* Healthy controls	0.142	0.409	0.039	0.563	0.409

Grade I: Well differentiated carcinoma; Grade II: Moderately differentiated carcinoma; Grade III: Poorly differentiated carcinoma; HDL-C: High density lipoprotein-Cholesterol; LDL-C: Low density lipoprotein-Cholesterol; VLDL: Very low density lipoprotein. P < 0.05 is significant.

**Table 3:** Comparison of lipid profile and tumour subsites between OSCC patients and healthy controls.

	Cholesterol	Triglyceride	HDL-C	LDL-C	VLDL
**Healthy Controls**	164.61 ± 29.11	104.08 ± 31.81	47.00 ± 9.50	90.58 ± 24.00	20.81 ± 6.36
**Tumour subsites**					
Oral cavity	125.50 ± 0.71	48.50 ± 0.71	30.50 ± 0.71	78.99 ± 1.41	8.90 ± 1.27
Tongue	115.20 ± 30.62	97.20 ± 36.39	22.2 ± 11.21	68.95 ± 19.98	19.44 ± 7.28
Mouth floor	125.50 ± 0.71	95.50 ± 0.71	25.50 ± 0.71	74.88 ± 1.24	18.60 ± 0.85
Lip/Buccal mucosa	131.78 ± 34.15	85.00 ± 30.37	24.67 ± 6.80	84.53 ± 25.34	17.00 ± 6.07
Maxilla	150.50 ± 23.33	49.50 ± 0.71	50.00 ± 9.90	85.06 ± 12.76	9.90 ± 0.14
Mandible	155.50 ± 31.82	99.50 ± 12.02	23.00 ± 8.50	105.64 ± 19.65	19.9 ± 2.40
Palate	114.50 ± 50.20	61.00 ± 31.11	19.50 ± 9.19	77.71 ± 32.64	12.20 ± 6.22
*p* value* comparison of lipid profile of OSCC and healthy controls					
Oral cavity * Healthy controls	0.021	0.019	0.021	0.430	0.012
Tongue * Healthy controls	0.001	0.881	0.000028	0.046	0.881
Mouth floor * Healthy controls	0.005	0.848	0.005	0.304	0.754
Lip/Buccal mucosa* Healthy controls	0.004	0.215	4.9E-7	0.413	0.215
Maxilla* Healthy controls	0.991	0.022	0.991	0.668	0.022
Mandible* Healthy controls	0.002	0.996	0.002	0.436	0.996
Palate* Healthy controls	0.001	0.079	0.001	0.409	0.079

HDL-C: High density lipoprotein-Cholesterol; LDL-C: Low density lipoprotein-Cholesterol; VLDL: Very low density lipoprotein. P < 0.05 is significant.

**Table 4:** Comparison of lipid profile and tumour stage between OSCC patients and healthy controls.

	Cholesterol	Triglyceride	HDL-C	LDL-C	VLDL
**Healthy Controls**	164.61 ± 29.11	104.08 ± 31.81	47.00 ± 9.50	90.58 ± 24.00	20.81 ± 6.36
**Tumour stage**					
Stage I	150.33 ± 34.99	90.67 ± 24.21	31.33 ± 6.81	94.63 ± 30.43	18.13 ± 4.84
Stage II	131.80 ± 35.94	82.40 ± 39.93	19.80 ± 5.58	89.62 ± 23.73	16.48 ± 7.98
Stage III	131.67 ± 24.28	81.33 ± 25.00	28.67 ± 6.25	81.37 ± 15.63	16.27 ± 5.00
Stage IV	111.17 ± 33.23	74.33 ± 24.83	22.17 ± 11.63	69.54 ± 24.75	14.87 ± 4.97
*p* value* comparison of lipid profile of OSCC and healthy controls					
Stage I* Healthy controls	0.327	0.613	0.008	0.874	0.613
Stage II* Healthy controls	0.019	0.267	0.000003	0.816	0.267
Stage III* Healthy controls	0.007	0.165	0.0001	0.284	0.165
Stage IV* Healthy controls	0.0002	0.058	0.00001	0.043	0.058

HDL-C: High density lipoprotein-Cholesterol; LDL-C: Low density lipoprotein-Cholesterol; VLDL: Very low density lipoprotein. P < 0.05 is significant.

**Table 5:** Correlation of ApoC1 with clinicopathological features of OSCC patients.

	Sample Size	Groups	ApoC1
Tumour Stage	20	Stage I (n = 3), Stage II (n = 5), Stage III (n = 6), Stage IV (n = 6)	ANOVA pval = 0.989
Tumour Grade	22	–	Pearson’s corr. coef. = 0.08, pval = 0.7
Tumour Size	20	–	Pearson’s corr. coef. = −0.095, pval = 0.673
Tumour Subsites	22	Oral cavity (n = 2), Tongue (n = 5), Mouth floor (n = 2), Lip/Buccal mucosa (n = 9), Maxilla (n = 2), Mandible (n = 2)	ANOVA pval = 0.13
